# SPP1-CD44-Mediated Crosstalk Between Endothelial and Macrophage Subpopulations Promotes Pancreatic Ductal Adenocarcinoma Progression Through S100A6 Upregulation

**DOI:** 10.3390/cells15201844

**Published:** 2026-10-11

**Authors:** Yanbin Zhou, Sixian Guo, Wenhu Zhou, Wei Wang

**Affiliations:** 1 Xiangya School of Pharmaceutical Sciences, Central South University, Changsha 410013, China; 2 School of Traditional Chinese Medicine, Shandong University of Traditional Chinese Medicine, Jinan 250355, China; 3 Faculty of Chinese Medicine, Medical Sciences Division, Macau University of Science and Technology, Macau 999078, China; 4 Hunan Key Laboratory of The Research and Development of Novel Pharmaceutical Preparations, School of Pharmaceutical Science, Changsha Medical University, Changsha 410219, China

**Keywords:** pancreatic ductal adenocarcinoma, endothelial cells, macrophages, cell–cell interaction, SPP1-CD44, S100A6

## Abstract

**Highlights:**

**What are the main findings?**

- Identification of pathogenic cell subsets and their enhanced crosstalk: Integrative analysis of single-cell RNA sequencing data identifies a distinct endothelial cell subpopulation (EC0) and a specific tumor-associated macrophage subset (Mac2). Intercellular communication between these two subsets is significantly augmented in the PDAC tumor microenvironment relative to paired adjacent normal tissue.
- S100A6 as a key mediator and prognostic indicator: The crosstalk between EC0 and Mac2 drives pronounced upregulation of S100A6, and elevated S100A6 expression is robustly associated with unfavorable prognosis in PDAC patients.

**What are the implications of the main findings?**

- Candidate prognostic biomarker for risk stratification: The S100A6-associated molecular signature identified in this study serves as a candidate prognostic biomarker for stratifying PDAC patients into high- and low-risk subgroups, providing supportive evidence for clinical patient management and reference for individualized treatment decision-making.
- Potential therapeutic target for PDAC intervention: Disrupting this pathogenic stromal communication axis, particularly targeting S100A6 and its downstream oncogenic effects, represents a promising novel therapeutic strategy for PDAC.

**Abstract:**

Pancreatic ductal adenocarcinoma (PDAC) progression is driven by complex cellular interactions within the tumor microenvironment (TME), where endothelial cell (EC)-tumor-associated macrophage (TAM) crosstalk significantly influences angiogenesis and immune modulation, although the specific subsets and consequences remain unclear. Here, we integrated four single-cell RNA sequencing datasets (50 PDAC; 21 normal adjacent tissues) to profile EC and macrophage subpopulations, subsequently employing CellChat to decipher intercellular communication networks and identify altered ligand–receptor interactions in PDAC. Correlation and Kaplan–Meier survival analyses (TCGA-PAAD) assessed associations and prognostic value, while functional validation used EdU, CCK-8, and Transwell assays on PDAC cells. We identified a distinct endothelial subset (EC0) and macrophage subset (Mac2) with enhanced interaction signals in PDAC. This EC0–Mac2 crosstalk correlated with significant S100A6 upregulation, which was associated with poor patient prognosis. Exogenous S100A6 treatment markedly enhanced PDAC cell proliferation and migration in vitro. In conclusion, EC-Mac crosstalk may promote PDAC progression via S100A6, and this interaction pattern warrants further investigation as a candidate prognostic biomarker and potential target for disrupting stromal communication.

## 1. Introduction

Pancreatic ductal adenocarcinoma (PDAC) remains one of the most lethal malignancies worldwide, with a dismal 5-year survival rate of approximately 10% despite extensive research efforts [1,2,3,4,5]. The extraordinary therapeutic resistance of PDAC stems largely from its unique tumor microenvironment (TME), which features an exceptionally dense desmoplastic stroma comprising up to 90% of the tumor mass [6,7,8]. This fibroinflammatory milieu creates a complex ecosystem where cellular crosstalk between malignant cells and stromal components is thought to drive tumor progression, metastasis, and treatment failure [9,10]. The current therapeutic landscape for PDAC is evolving, with standard-of-care regimens, including modified FOLFIRINOX or gemcitabine-based therapies, and emerging targeted strategies, such as KRAS G12C inhibitors [11], alongside novel approaches targeting the TME, including FAK inhibitors, which are also under active investigation [12]. Among these stromal elements, the reciprocal interactions between vascular endothelial cells (ECs) and tumor-associated macrophages (TAMs) have emerged as a critical axis in PDAC pathogenesis, though the specific cellular subsets and molecular mechanisms involved remain incompletely characterized [9,13,14].

Recent advances in single-cell technologies have revolutionized our understanding of cellular heterogeneity within the PDAC TME [10]. High-resolution transcriptomic analyses have identified multiple functionally distinct subpopulations of both ECs and TAMs, each exhibiting unique gene expression signatures and spatial distributions. ECs in PDAC demonstrate remarkable plasticity, with subsets specialized for angiogenesis, immune regulation, or metabolic support [13,15]. Similarly, TAMs display a spectrum of activation states ranging from pro-inflammatory to immunosuppressive phenotypes [16]. Spatial transcriptomic studies have revealed non-random colocalization patterns between specific EC and TAM subsets, suggesting the existence of specialized cellular niches that may influence tumor progression through paracrine signaling networks [17,18].

The S100 protein family, comprising 21 known calcium-binding members, has gained increasing attention for its role in mediating intercellular communication within tumor microenvironments [19,20]. These proteins function as damage-associated molecular patterns (DAMPs) and can be secreted to activate various cell surface receptors, including RAGE and TLRs [21]. In PDAC, several S100 proteins show markedly elevated expression compared to normal pancreatic tissue and have been associated with poor clinical outcomes [22]. Mechanistically, S100 proteins have been implicated in diverse oncogenic processes including epithelial–mesenchymal transition, extracellular matrix remodeling, and immune cell recruitment [19]. However, the precise cellular sources of these proteins within the PDAC TME and the regulatory networks controlling their expression remain poorly understood, representing a significant knowledge gap in the field.

Against this research backdrop, this study was specifically designed to investigate the cellular and molecular basis of endothelial–macrophage communication in PDAC, and elucidate how this core intercellular crosstalk modulates the expression, secretion and oncogenic functions of S100 proteins in the PDAC tumor microenvironment. By integrating single-cell transcriptomics analysis with systematic functional validation, we aimed to comprehensively characterize the heterogeneity of endothelial cell (EC) and tumor-associated macrophage (TAM) subpopulations in PDAC and further identify and verify key intercellular signaling pathways derived from endothelial–macrophage interactions that govern the regulatory networks of S100 proteins. We additionally explored the downstream oncogenic effects of the EC-TAM crosstalk-mediated S100 protein dysregulation on malignant progression of PDAC. Collectively, our findings provide in-depth insights into the complex cellular ecosystems driving PDAC pathogenesis and nominate critical S100 family candidate molecules for future mechanistic and translational functional studies.

## 2. Materials and Methods

### 2.1. Integration and Quality Control of Single-Cell RNA Sequencing Data

We systematically integrated four independent single-cell RNA sequencing datasets (GSE155698 [23], GSE197177 [24], GSE212966 [25], CRA001160 [26]) encompassing 50 PDAC tumor samples and 21 matched adjacent normal tissues from the NCBI GEO and CNCB (National Center for Biotechnology, China) databases. The raw sequencing data were preprocessed with Seurat (version 5.1) following rigorous quality control protocols, which included the following steps: (1) cells expressing fewer than 200 genes were excluded to remove low-quality cells; (2) cells with a mitochondrial gene proportion greater than 20% were removed to exclude stressed or apoptotic cells; (3) potential doublets were identified and removed using DoubletFinder (v2.0) with an expected doublet rate of 8%. Data normalization was conducted via the SCTransform method, selecting the top 3000 highly variable genes identified using variance stabilization transformation. To address batch effects between different datasets, we applied Harmony integration (v0.1.0) with default parameters, which successfully aligned cells across different batches while preserving biological heterogeneity.

### 2.2. Endothelial and Macrophage Subpopulation Analysis

Endothelial cells were stringently defined by the co-expression of the established markers CD31 (PECAM1) and VWF, while macrophages were defined by expression of CD68 and CD163. For subpopulation analysis, we performed UMAP dimensionality reduction using the first 20 principal components determined by elbow plot analysis. The FindNeighbors and FindClusters functions were applied with a resolution parameter of 0.1 to optimize cluster separation. Differential gene expression analysis between subpopulations was performed using the Wilcoxon rank-sum test with Bonferroni correction, with significance thresholds set at log2 fold change > 0.25 and adjusted *p*-value < 0.05. Marker genes specific to each cluster were cross-referenced against established cell type signatures in the CellMarker database.

### 2.3. Cell–Cell Interaction Network Analysis

Cell–cell communication analysis was performed using CellChat (v1.1.3) with its comprehensive ligand–receptor interaction database containing 2021 validated pairs [27,28]. The analysis pipeline included: (1) calculation of communication probabilities for all possible ligand–receptor pairs; (2) statistical evaluation using 1000 permutations to determine significant interactions (*p* < 0.01 after false discovery rate correction); (3) quantification of information flow by computing the sum of communication probabilities for each signaling pathway; (4) comparative analysis between tumor and ADJ tissues to identify PDAC-specific interactions. Special attention was given to interactions between endothelial subpopulation and macrophage subpopulation, which showed the most significant changes in PDAC.

### 2.4. Gene Expression Correlation Analysis

We performed systematic correlation analyses to investigate relationships between key genes involved in the EC0–Mac2 interaction axis. Pearson correlation coefficients were calculated for: (1) CD44 expression in EC0 versus SPP1 expression in Mac2 across all samples; (2) SPP1 expression versus S100A6 expression across macrophage subpopulations. The statistical significance of correlations was assessed using a t-distribution, with *p* < 0.05 considered significant. Correlation networks were visualized using Cytoscape (v3.8.2), with edge weights representing correlation strength and node sizes reflecting average expression levels.

### 2.5. Cell Culture and Treatment

PANC-1 human pancreatic cancer cells (ATCC CRL-1469), MIA PaCa-2 (CL-0627, Procell) and BxPC-3 cells (CL-0042, Procell) were cultured in high-glucose DMEM supplemented with 10% fetal bovine serum (FBS), 2 mM L-glutamine, and 1% penicillin-streptomycin. Cells were maintained at 37 °C in a humidified 5% CO_2_ atmosphere. Authentication was performed by short tandem repeat (STR) profiling, and cells were routinely tested for mycoplasma contamination using the MycoAlert Mycoplasma Detection Kit (Lonza, Shanghai, China). For experiments, cells between passages 5 and 20 were used, and medium was refreshed every 2–3 days. Cell confluence was monitored daily, and cells were passaged at 80–90% confluence using 0.25% trypsin-EDTA.

For functional assays, cells were treated with recombinant human S100A6 protein (Proteintech, Wuhan, China, cat. no. Ag0385) at a concentration of 0.25 µg/μL. Treatment durations were as follows: 48 h for proliferation assays (CCK-8 and EdU) and 24 h for migration assays (Transwell). Control cells received an equal volume of vehicle (PBS) without the recombinant protein.

### 2.6. SPP1 Knockdown and Rescue in the EC–MAC Co-Culture System

PMA-differentiated THP-1 macrophages were transfected with SPP1-targeting siRNA (si-SPP1) or scrambled negative-control siRNA using Lipofectamine 3000 (Invitrogen, Carlsbad, CA, USA) according to the manufacturer’s instructions, and knockdown efficiency was verified by qRT-PCR. Transfected macrophages were co-cultured with HUVECs at a macrophage-to-EC ratio of 1:5 for 48 h. Six experimental groups were established: (1) control macrophages + EC (Ctrl); (2) SPP1-knockdown macrophages + EC (si-SPP1); (3) control macrophages + EC + anti-CD44 neutralizing antibody; (4) control macrophages + EC + IgG isotype control; (5) SPP1-knockdown macrophages + EC + recombinant human SPP1 (rSPP1); and (6) SPP1-knockdown macrophages + EC + rSPP1 + anti-CD44 neutralizing antibody. After 48 h of co-culture, cells were harvested, and S100A6 mRNA expression in the co-culture system was quantified by qRT-PCR as described below.

### 2.7. Quantitative PCR Analysis

Total RNA was isolated from cells with TRIzol reagent according to the manufacturer’s instructions. The concentration and purity of the isolated RNA were determined using a NanoDrop spectrophotometer (Thermo Fisher Scientific, Wilmington, DE, USA), with an A260/A280 ratio > 1.8 considered acceptable. cDNA synthesis was performed with 1 μg total RNA using the PrimeScript RT reagent kit (TaKaRa, Shiga, Japan) with gDNA Eraser. Quantitative PCR amplification was performed on a QuantStudio 6 system using SYBR Green Master Mix (TakaRa, Japan), under the following thermal cycling conditions: initial denaturation at 95 °C for 30 s, followed by 40 cycles of 95 °C for 5 s and 60 °C for 30 s. GAPDH served as the internal control, and relative gene expression was calculated using the 2-ΔΔCt method. Primer sequences were as follows: S100A6 forward 5′-GCTGGAGGTGCTAGCAAGAA-3′, reverse 5′-TCCTCCTCCTCCTTGTCCTT-3′; GAPDH forward 5′-GGAGCGAGATCCCTCCAAAAT-3′, reverse 5′-GGCTGTTGTCATACTTCTCATGG-3′.

### 2.8. EDU Proliferation Assay

Cell proliferation was assessed using the Click-iT Plus EDU Alexa Fluor 647 Imaging Kit (Thermo Fisher, Waltham, MA, USA). Briefly, cells were seeded into 24-well plates at a density of 5 × 10^4^ cells/well and cultured overnight to facilitate attachment. EDU labeling solution (10 μM final concentration) was added to the culture medium for 2 h at 37 °C. Cells were then fixed with 4% paraformaldehyde for 15 min, permeabilized with 0.5% Triton X-100 (Beyotime, Shanghai, China) for 20 min, and stained with the Click-iT reaction cocktail for 30 min, protected from light. Nuclei were counterstained with Hoechst 33342 (1 μg/mL) (Beyotime, Shanghai, China) for 10 min. Fluorescence microscopy was performed on a Zeiss Axio Observer microscope (20× objective) (Axio Observer 3, Jena, Germany), with five random fields per well quantified in ImageJ (V1.54). The proliferation index was calculated as the percentage of EDU-positive cells among total Hoechst-positive cells.

### 2.9. CCK-8 Cell Viability Assay

The viability of cells was assessed via the CCK-8 assay in accordance with the manufacturer’s instructions. Cells were seeded in 96-well plates at 3 × 10^3^ cells/well in 100 μL complete medium. At designated time points (0, 24, 48, 72, and 96 h), 10 μL of CCK-8 reagent was added to each well, followed by incubation at 37 °C for 2 h. Optical density (OD) was measured at 450 nm with a microplate reader, and readings at 650 nm were used as a reference. Background absorbance from blank wells containing medium only was subtracted from all measurements. Each condition was performed in six replicates, and results were normalized to the 0 h time point to calculate fold changes in viability.

### 2.10. Transwell Migration Assays

Cell migration assays were performed by seeding 5 × 10^4^ cells in 200 μL serum-free medium into the upper chambers of Transwell inserts with 8 μm pores (Corning, New York, NY, USA). The lower chambers contained 600 μL medium with 10% FBS as a chemoattractant. After 24 h incubation at 37 °C, non-migrated cells on the upper surface were removed with cotton swabs. Migrated cells on the lower surface were fixed with 4% paraformaldehyde for 15 min and stained with 0.1% crystal violet for 20 min. Five random fields per membrane were imaged at 10× magnification and counted using ImageJ. Results were expressed as the average number of migrated cells per field.

### 2.11. ELISA Assays

The secretion level of S100A6 in cell culture supernatant was quantified via ELISA. A co-culture system of PMA-differentiated THP-1 macrophages and HUVECs was used for all treatments, with all cell culture procedures performed at 37 °C in a 5% CO_2_ humidified incubator. Two parallel sets of bidirectional perturbation assays were conducted in the EC-MAC co-culture system. In the first assay set, the co-culture system was pre-stimulated with recombinant CD44 protein for 24 h, followed by medium refreshment and subsequent 24 h incubation with SPP1 neutralizing antibody to block endogenous SPP1 signaling. In the second parallel assay set, cells were pre-treated with recombinant SPP1 protein for 24 h, after which the culture medium was replaced, and cells were further incubated with CD44 neutralizing antibody for an additional 24 h. Following all treatments, S100A6 protein concentration in the culture supernatant was quantified for each group separately.

After treatment, culture supernatants from all groups were collected and centrifuged to eliminate cell debris. S100A6 concentrations in supernatants were measured using a commercial human S100A6 ELISA kit (Proteintech, Wuhan, China) in accordance with the manufacturer’s protocols. Sample absorbance was detected with a microplate reader, and S100A6 levels were calculated based on the standard curve for subsequent statistical analysis.

### 2.12. Western Blot Analysis

Total protein was extracted using RIPA buffer supplemented with protease and phosphatase inhibitors. Protein concentration was determined using the BCA assay. In total, 30 μg of protein per sample was separated on 12% SDS-PAGE gels and electrotransferred to PVDF membranes. Membranes were blocked with 5% non-fat milk for 1 h and then probed overnight at 4 °C with primary antibodies against S100A6 (1:1000, Abcam ab124805, Waltham, MA, USA) and β-actin (1:5000, Sigma A5441. St. Louis, MO, USA). Following three washes with TBST, membranes were incubated with HRP-conjugated secondary antibodies (1:5000) for 1 h at room temperature. Protein bands were visualized using enhanced chemiluminescence (ECL) substrate and captured with a ChemiDoc imaging system. Band intensities were quantified using Image Lab software (v1.2), with S100A6 expression normalized to β-actin.

### 2.13. Clinical Sample Collection and Ethics Statement

A total of 3 PDAC tissue samples and 3 matched adjacent non-tumor pancreatic tissue samples were obtained from patients who underwent surgical resection. All samples were histologically confirmed by two independent pathologists. Immediately after resection, tissue specimens were snap-frozen in liquid nitrogen and stored at −80 °C until use for quantitative real-time PCR (qPCR) and Western blot analysis. The study was conducted in accordance with the Declaration of Helsinki and was approved by the Institutional Ethics Committee of Xiangya Pharmaceutical Sciences School of Central South University, China (approval no. 2026-28, 20 November 2025). Written informed consent was obtained from all patients prior to sample collection.

### 2.14. Survival Analysis

Clinical data and RNA-seq expression profiles for the TCGA-PAAD cohort (*n* = 177) were obtained from the UCSC Xena browser (v2.99). Patients were stratified into high and low expression groups based on median expression of target genes. Kaplan–Meier survival curves were generated, and statistical significance was assessed using the log-rank test. Univariate and multivariate Cox proportional hazards models were constructed to evaluate the prognostic value of gene signatures while adjusting for age, gender, and tumor stage. Hazard ratios with 95% confidence intervals were calculated. All survival analyses were performed using R (v4.1.0) with the survival and survminer packages (version 0.5.2).

### 2.15. Statistical Analysis

All experiments were performed with at least three independent biological replicates. Data are presented as mean ± standard error of the mean (SEM). For comparisons between two groups, two-tailed Student’s t-tests were used. Multiple group comparisons were analyzed by one-way ANOVA followed by Tukey’s post hoc test. For all statistical tests, *p* < 0.05 was considered statistically significant. All analyses were performed using GraphPad Prism (v9.0) and R (v4.1.0). Data visualization was created using ggplot2 (v3.3.5) and ComplexHeatmap (v2.8.0) packages in R.

## 3. Results

### 3.1. Endothelial Subpopulation Heterogeneity and EC0 Expansion in PDAC

**Figure 1:**
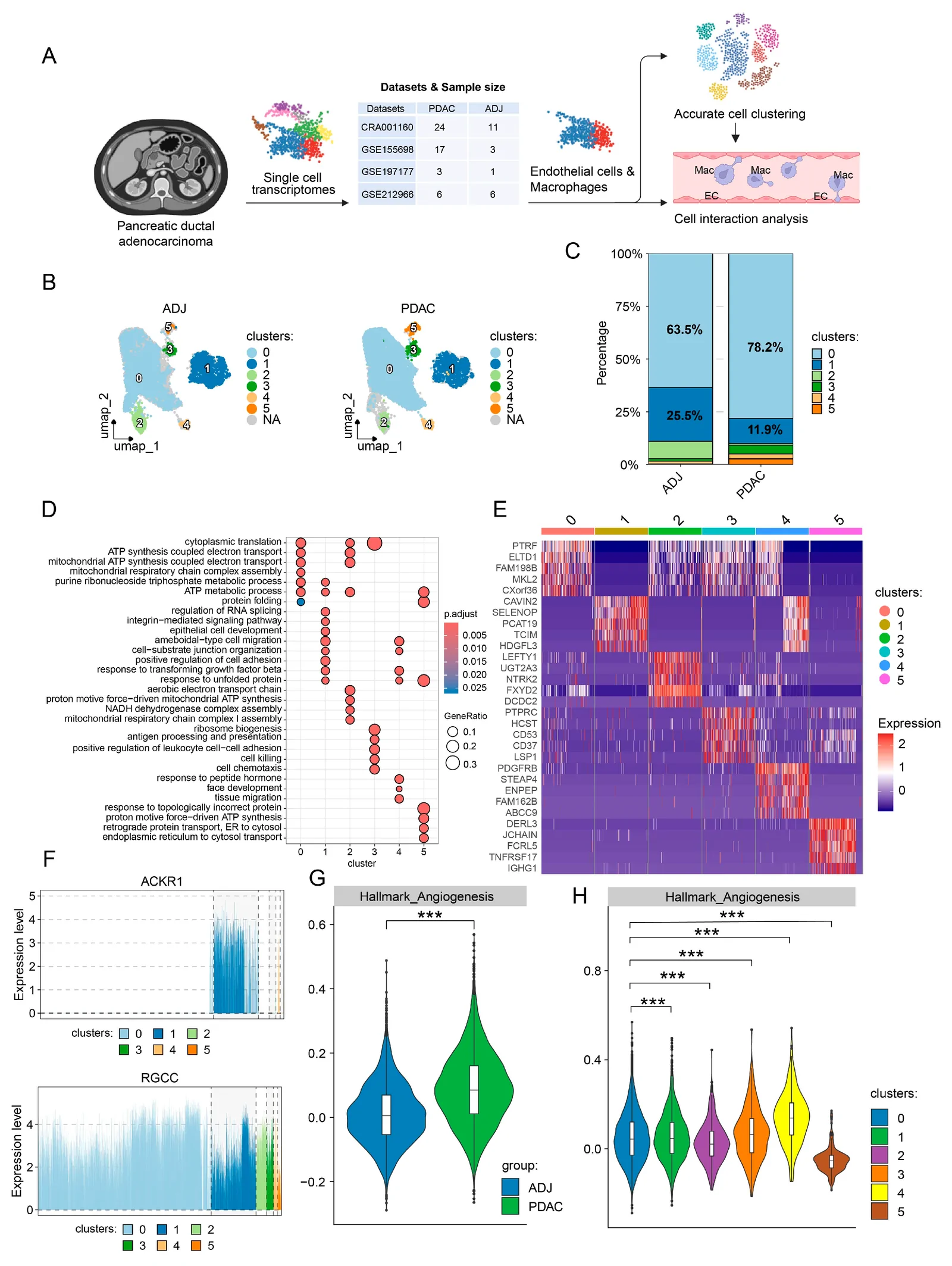
Characterization of endothelial subpopulations in PDAC. (**A**) Schematic workflow of the study. Single-cell transcriptomic datasets from PDAC (50 samples) and adjacent normal (ADJ, 21 samples) tissues were integrated, followed by cell clustering, endothelial/macrophage isolation, and cell interaction analysis. (**B**) UMAP visualization of endothelial cell subpopulations in ADJ and PDAC tissues. Clusters are color-coded (0–5). (**C**) Proportion of endothelial subpopulations in ADJ and PDAC. EC0 is significantly expanded in PDAC (ADJ: 63.5% vs. PDAC: 78.2%). (**D**) Functional enrichment analysis of EC0 in PDAC. Dot size represents gene ratio; color indicates adjusted *p*-value. Pathways related to angiogenesis and metabolism are enriched. (**E**) Heatmap of top 50 differentially expressed genes in endothelial subpopulations. Expression levels are scaled by row (red: high; blue: low). (**F**) Expression of endothelial markers (ACKR1, RGCC) across subpopulations. (**G**) Violin plots comparing “Hallmark_Angiogenesis” pathway activity between ADJ and PDAC. *** *p* < 0.001. (**H**) Pathway activity scores of “Hallmark_Angiogenesis” across endothelial subpopulations in PDAC.

To systematically characterize endothelial cell (EC) heterogeneity in pancreatic ductal adenocarcinoma (PDAC), we integrated single-cell RNA sequencing data from four independent datasets (GSE155698, GSE197177, GSE212966, and CRA001160), encompassing 50 PDAC tissues and 21 adjacent normal (ADJ) tissues (Figure 1A). Endothelial cells were first isolated based on canonical markers (FLT1, PECAM1/CD31, and VWF), with validation across all datasets confirming robust expression of endothelial signatures (Supplementary Figure S1). Unsupervised clustering of ECs identified six distinct subpopulations (EC0–EC5) in both ADJ and PDAC tissues (Figure 1B).

Notably, the proportion of EC subpopulations differed drastically between ADJ and PDAC. In ADJ tissues, EC0 constituted 63.5% of total ECs, whereas in PDAC, EC0 expanded to 78.2%, becoming the dominant endothelial subpopulation (Figure 1C). To characterize EC0, we performed differential gene expression analysis, which revealed upregulation of pro-angiogenic genes (e.g., ELTD1) and metabolic regulators (e.g., MKL2) compared to other clusters (Figure 1E). Functional enrichment analysis further supported a pro-angiogenic and metabolically active phenotype, with EC0 enriched in pathways such as “ATP synthesis coupled electron transport,” “mitochondrial respiratory chain complex assembly,” and “regulation of angiogenesis” (Figure 1D).

Key markers of EC0 included RGCC (regulator of cell cycle), which was specifically upregulated in this subpopulation, whereas ACKR1 (atypical chemokine receptor 1) expression was not detected in EC0 (Figure 1F). To quantify angiogenic activity, we assessed “Hallmark_Angiogenesis” pathway scores, which were significantly higher in PDAC ECs compared to ADJ (Figure 1G; **** *p* < 0.0001). Among subpopulations, EC0 exhibited the highest angiogenic activity, followed by EC4 and EC3 (Figure 1H), confirming its central role in PDAC vascular remodeling.

To validate these findings, we performed pairwise differential expression analysis between EC subpopulations, confirming EC0-specific upregulation of angiogenic effectors and downregulation of quiescent endothelial markers (Supplementary Figure S2A). Pathway enrichment of “Sprouting Angiogenesis,” “EC Migration,” and “EC Proliferation” was significantly higher in PDAC ECs, with EC0 showing the strongest activation of these pathways (Supplementary Figure S2B,C). Collectively, these data identify EC0 as an expanded, pro-angiogenic endothelial subpopulation in PDAC, suggesting its critical role in tumor vascularization.

### 3.2. Macrophage Subpopulation Heterogeneity and Mac2 Expansion in PDAC

**Figure 2:**
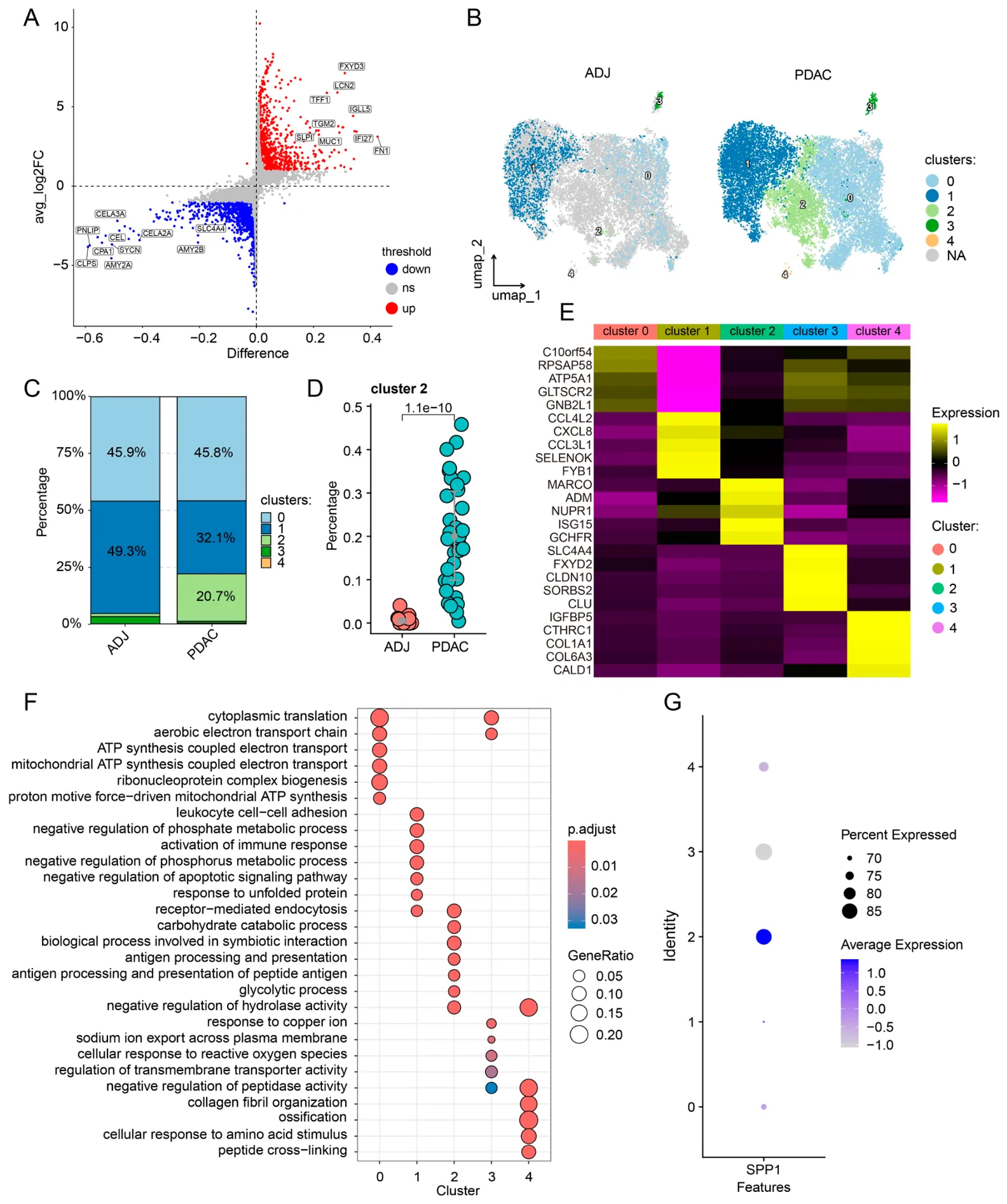
Macrophage subpopulation heterogeneity and Mac2 enrichment in PDAC. (**A**) Volcano plot showing global transcriptomic differences between PDAC and adjacent normal (ADJ) macrophages. Red dots indicate upregulated genes (log2FC > 0.25, adjusted *p* < 0.05); blue dots indicate downregulated genes (log2FC < −0.25, adjusted *p* < 0.05). Selected pro-tumorigenic and inflammatory genes are labeled. (**B**) UMAP visualization of macrophage subpopulations in ADJ and PDAC tissues. Clusters are color-coded (0–4), with NA indicating unassigned cells. (**C**) Bar plot showing proportional distribution of macrophage subpopulations in ADJ and PDAC tissues. (**D**) Scatter plot quantifying the percentage of Mac2 (cluster 2) in ADJ versus PDAC tissues (ADJ: 0.1% vs. PDAC: 32.1%). (**E**) Heatmap of top 50 differentially expressed genes across macrophage subpopulations (Mac0–Mac4). Expression levels are scaled by row (yellow: high; purple: low). (**F**) Functional enrichment analysis of macrophage subpopulations in PDAC. Dot size represents gene ratio; color indicates adjusted *p*-value. (**G**) Dot plot showing SPP1 expression across macrophage subpopulations. Mac2 exhibits the highest average expression and percentage of positive cells (85%).

To characterize macrophage heterogeneity in PDAC, we analyzed transcriptomic profiles of macrophages (defined by CD68 + CD163+) isolated from the same integrated datasets. Global differential gene expression analysis between PDAC and ADJ macrophages revealed significant upregulation of pro-tumorigenic genes (LCN2 and FN1) and downregulation of inflammatory markers (CLPS) in PDAC (Figure 2A). Unsupervised clustering identified five distinct macrophage subpopulations (Mac0–Mac4) with differential distribution between ADJ and PDAC tissues (Figure 2B).

Notably, the proportion of subpopulations varied significantly between ADJ and PDAC. In ADJ tissues, Mac0 (49.3%) and Mac1 (46.6%) were the dominant subsets, whereas in PDAC, Mac2 emerged as the most prevalent subpopulation (32.1%), accompanied by a corresponding decrease in Mac0 (20.7%) (Figure 2C). Quantitative analysis confirmed a significant expansion of Mac2 in PDAC compared to ADJ (ADJ: 0.1% vs. PDAC: 32.1%; Figure 2D).

Differential gene expression analysis of Mac2 revealed upregulation of immunosuppressive markers (MARCO, ADM), matrix remodeling enzymes, and metabolic regulators (SCD1, ACSL1) (Figure 2E). Functional enrichment analysis further supported a pro-tumorigenic phenotype, with Mac2 enriched in pathways related to antigen processing and presentation and the glycolytic process (Figure 2F).

Notably, Mac2 exhibited the highest expression of SPP1 (osteopontin) among all macrophage subpopulations, with 85% of Mac2 cells expressing SPP1 at significantly higher levels compared to other subsets (Figure 2G). Given the established role of SPP1 in tumor progression and immune suppression, these findings suggest Mac2 may promote PDAC pathogenesis through SPP1-mediated signaling. Collectively, our data identify Mac2 as a PDAC-enriched macrophage subpopulation with a pro-tumorigenic, immunosuppressive phenotype, highlighting its potential as a therapeutic target.

### 3.3. Enhanced Mac2-EC0 Communication via the SPP1-CD44 Axis in PDAC

**Figure 3:**
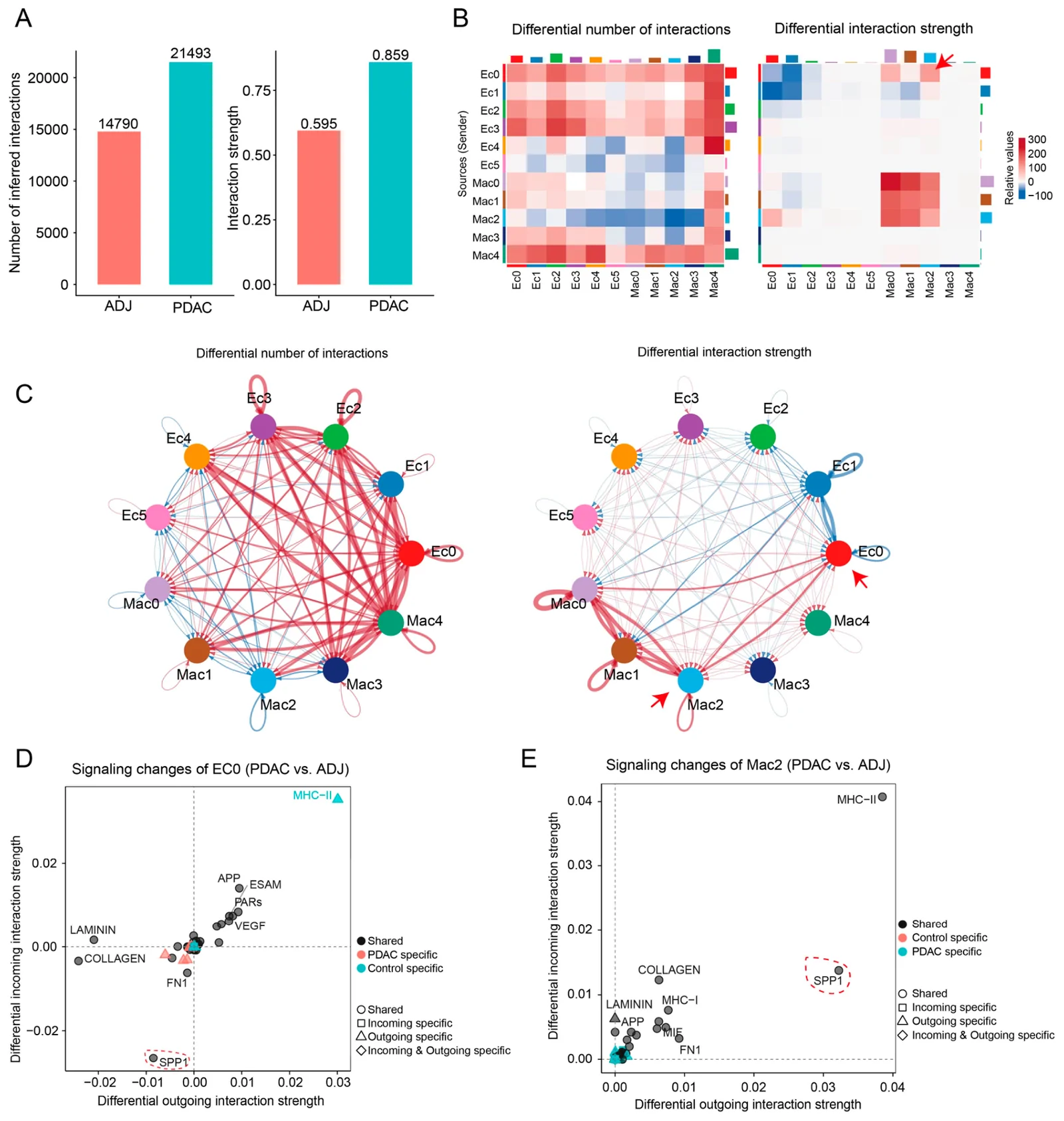
Cell–cell interaction networks between endothelial and macrophage subpopulations in PDAC. (**A**) Bar plots showing total number of inferred interactions (**left**) and average interaction strength (**right**) between endothelial and macrophage subpopulations in control (ADJ) and PDAC tissues. (**B**) Heatmaps comparing differential number of interactions (**left**) and interaction strength (**right**) between endothelial (EC0–EC5) and macrophage (Mac0–Mac4) subpopulations in PDAC versus control. Red/blue colors indicate increased/decreased interactions in PDAC. (**C**) Network diagrams visualizing differential number of interactions (**left**) and interaction strength (**right**) between endothelial and macrophage subpopulations. EC0 (red node) and Mac2 (blue node) show strengthened connections in PDAC (red edges). (**D**) Scatter plot showing differential outgoing vs. incoming interaction strength for EC0 in PDAC vs. control. SPP1 signaling is highlighted as a PDAC-specific incoming pathway. (**E**) Scatter plot showing differential outgoing vs. incoming interaction strength for Mac2 in PDAC vs. control. SPP1 signaling is highlighted as a PDAC-specific outgoing pathway.

To investigate intercellular communication between endothelial and macrophage subpopulations, we performed CellChat analysis on the integrated datasets. PDAC tissues exhibited a 44.2% increase in total inferred cell–cell interactions compared to ADJ and a 44.4% higher average interaction strength (Figure 3A). Focused analysis of endothelial–macrophage interactions revealed that EC0 and Mac2 exhibited the most significant gains in both interaction number and strength in PDAC (Figure 3B). Network visualization confirmed that EC0 and Mac2 formed a central communication hub in PDAC, with more connections and higher interaction strength compared to ADJ (Figure 3C).

Signaling pathway analysis of EC0 in PDAC identified upregulation of pro-angiogenic pathways, including VEGF signaling, together with increased incoming SPP1 signaling (Figure 3D). Conversely, Mac2 in PDAC showed increased outgoing signaling through SPP1, COLLAGEN and MHC-II pathways (Figure 3E), consistent with Mac2 being the SPP1-sending population and EC0 the CD44-expressing recipient population. Global pathway enrichment analysis further confirmed that SPP1 signaling exhibited the highest relative information flow in PDAC compared to all other pathways (Supplementary Figure S3A), with EC0–Mac2 interactions accounting for 37.6% of total SPP1-mediated communication (Supplementary Figure S3B,C).

**Figure 4:**
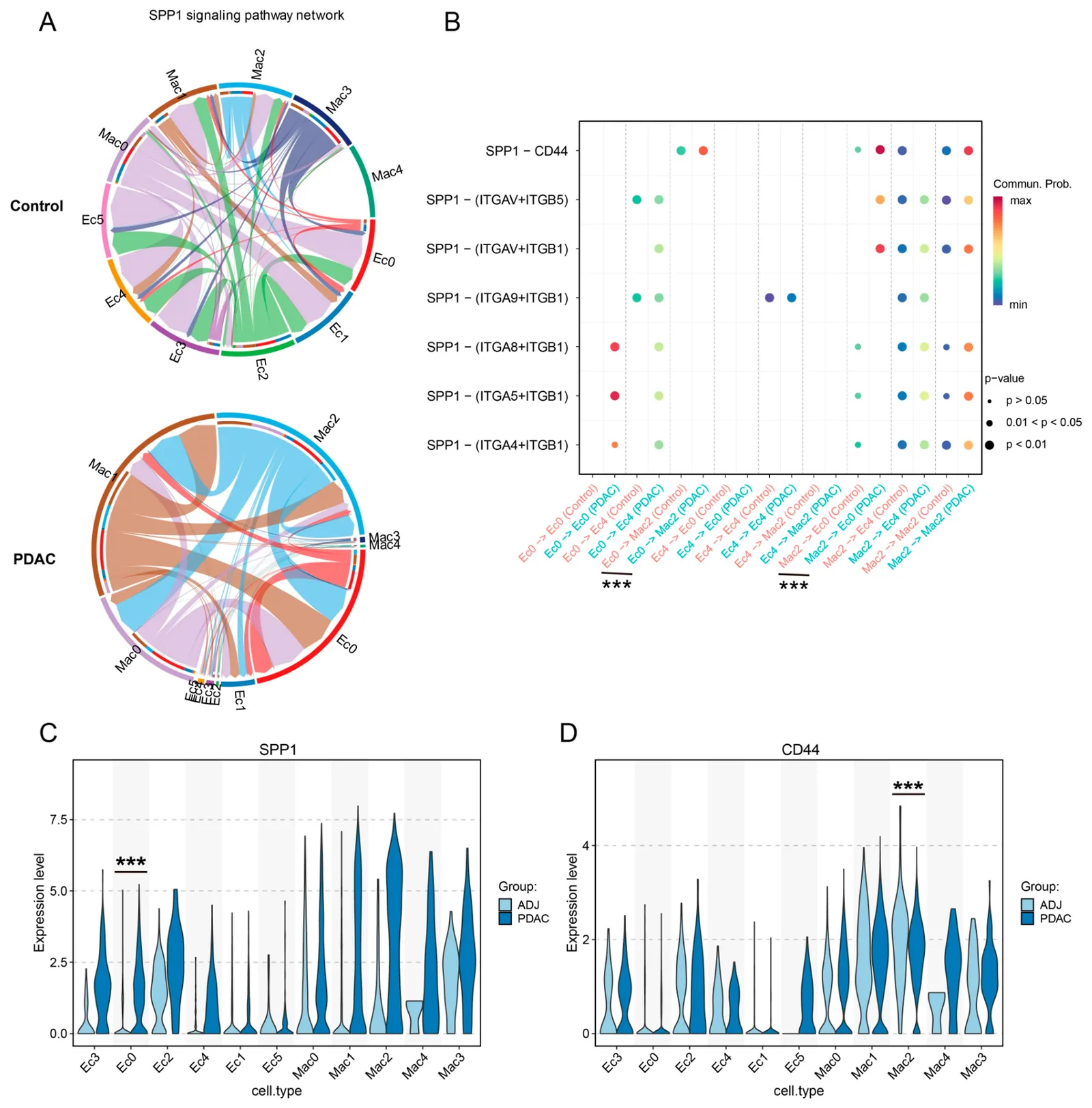
SPP1-CD44 signaling axis between Mac2 and EC0 in PDAC. (**A**) Circular network diagrams showing SPP1 signaling pathways in control (ADJ, top) and PDAC (bottom) tissues. Arrows indicate directional ligand–receptor interactions between endothelial (EC0–EC5) and macrophage (Mac0–Mac4) subpopulations. PDAC diagram highlights enhanced SPP1 signaling from Mac2 (blue) to EC0 (red). (**B**) Dot plots showing significance (−log10 *p*-value) and interaction probability of SPP1 ligand–receptor pairs. The SPP1-CD44 axis exhibits the highest significance (*** *p* < 0.001) and interaction probability in PDAC. (**C**) Violin plots showing CD44 expression across endothelial and macrophage subpopulations in ADJ and PDAC. EC0 exhibits the highest CD44 expression in PDAC (*** *p* < 0.001). (**D**) Violin plots showing SPP1 expression across endothelial and macrophage subpopulations in ADJ and PDAC. MAC2 exhibits the highest SPP1 expression in PDAC (*** *p* < 0.001).

To characterize the SPP1 signaling axis, we analyzed ligand–receptor pairs and found that the SPP1-CD44 interaction exhibited the highest significance (*p* < 0.001) and interaction probability in PDAC (Figure 4B). Circular network diagrams visualized the dramatic remodeling of SPP1 signaling in PDAC, with Mac2 emerging as the primary source of SPP1 and EC0 as the dominant recipient (Figure 4A). Expression validation confirmed that SPP1 was specifically upregulated in Mac2 and CD44 (the primary SPP1 receptor) was most highly expressed in EC0 in PDAC tissues (Figure 4C,D).

Dimensionality reduction in signaling pathways further separated PDAC from ADJ based on SPP1-CD44 axis activation, with EC0 and Mac2 clustering in a distinct PDAC-specific signaling space (Supplementary Figure S3D). These findings were validated by correlation analysis, which showed a strong positive correlation between SPP1 expression in Mac2 and CD44 expression in EC0 in PDAC (r = 0.68, * *p* < 0.001), but not in ADJ (r = 0.12, *p* = 0.34). Collectively, these results identify the SPP1-CD44 signaling axis as a key mediator of Mac2-EC0 communication in PDAC, representing a potential therapeutic target for disrupting tumor angiogenesis.

### 3.4. S100A6 Promotes PDAC Progression and Predicts Poor Prognosis

**Figure 5:**
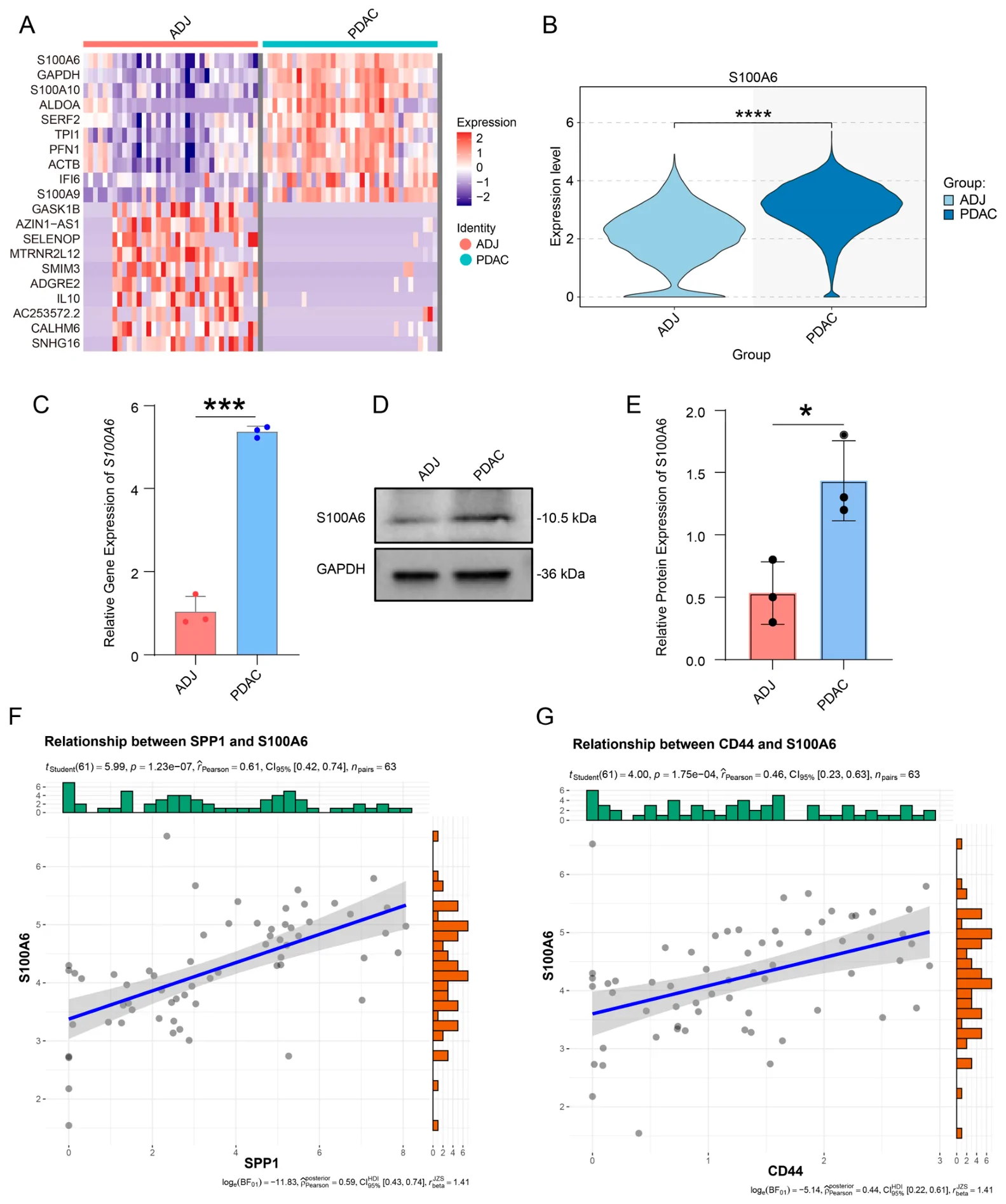
S100A6 is upregulated in PDAC and correlates with the SPP1-CD44 axis. (**A**) Heatmap of differentially expressed genes in Mac2 between PDAC and adjacent normal (ADJ) tissues, with S100A6 highlighted. Expression levels are scaled by row (red: high; purple: low). (**B**) Violin plot showing S100A6 expression in ADJ and PDAC tissues in scRNA datasets (**** *p* < 0.0001). (**C**) qPCR validation of S100A6 mRNA expression in ADJ and PDAC tissues (*** *p* < 0.0001). (**D**) Western blot analysis of S100A6 protein expression in ADJ and PDAC tissues. GAPDH served as a loading control. (**E**) Quantification of S100A6 protein expression from Western blot (*n* = 3 per group; * *p* < 0.05). (**F**) Correlation analysis between SPP1 and S100A6 expression in PDAC tissues (r = 0.69, *p* < 0.0001). (**G**) Correlation analysis between CD44 and S100A6 expression in PDAC tissues (r = 0.61, *p* < 0.0001).

To identify downstream effectors of the Mac2-EC0 SPP1-CD44 axis, we analyzed differentially expressed genes in Mac2 and identified S100A6 (a calcium-binding protein) as one of the most upregulated genes in PDAC (log2FC = 1.86, adjusted *p* < 0.001; Figure 5A). Validation in clinical samples confirmed that S100A6 expression was significantly higher in PDAC tissues compared to ADJ at both mRNA (Figure 5C; *p* < 0.0001) and protein levels (Figure 5D,E; *p* < 0.05). Notably, Pearson correlation analysis revealed strong positive correlations between S100A6 and both SPP1 (r = 0.69, * *p* < 0.0001) and CD44 (r = 0.61, * *p* < 0.0001) expression in PDAC (Figure 5F,G), suggesting S100A6 may be a downstream target of the SPP1-CD44 axis.

**Figure 6:**
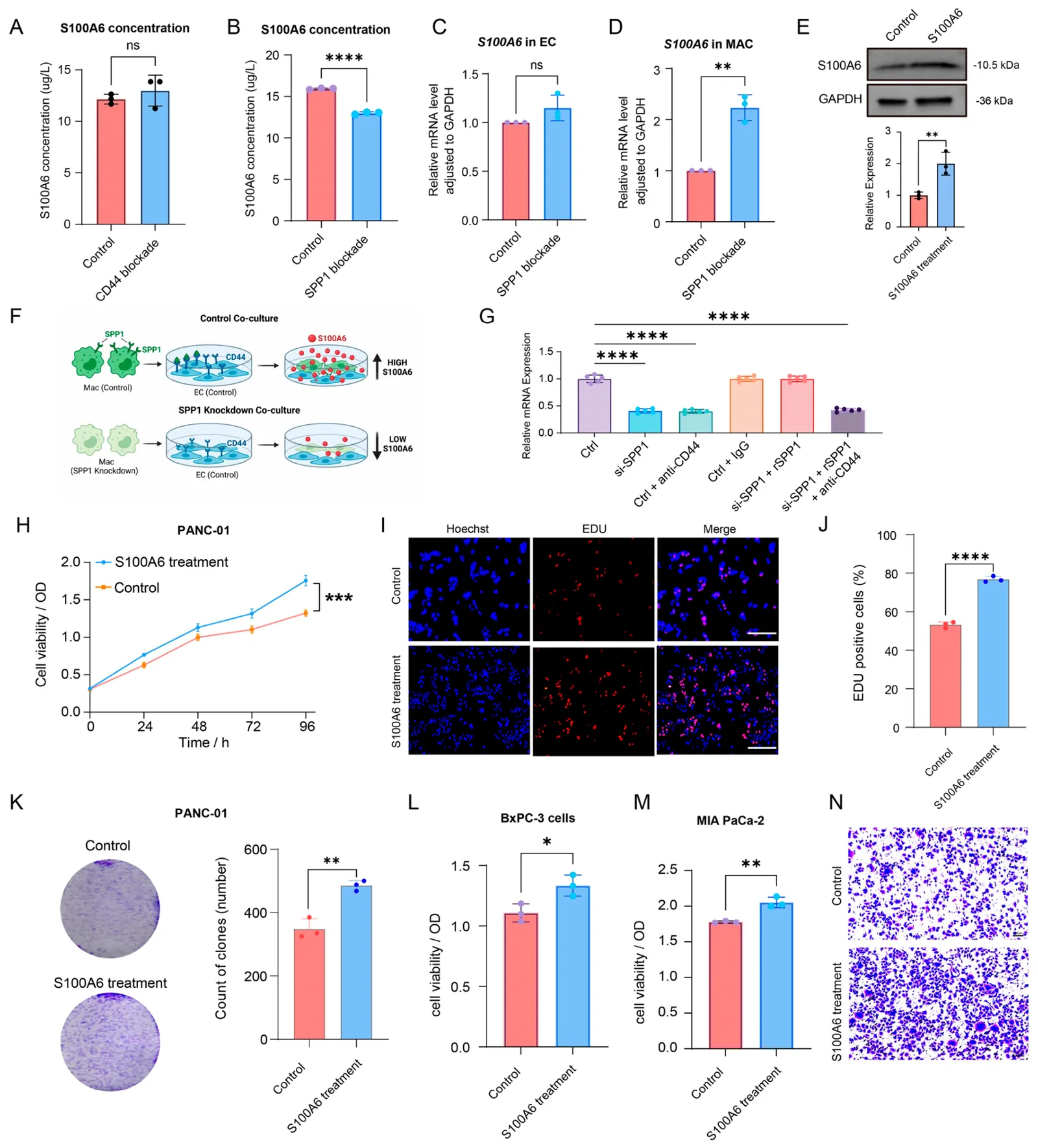
SPP1-CD44 signaling induces S100A6 expression, and S100A6 promotes proliferation and migration of pancreatic ductal adenocarcinoma (PDAC) cells. (**A**) S100A6 protein concentration in the culture supernatant of the EC-MAC co-culture system and recombinant SPP1 stimulation with or without CD44 blockade, detected by ELISA (ns, no significant difference). (**B**) S100A6 protein concentration in the culture supernatant of the EC-MAC co-culture system and recombinant CD44 stimulation with or without SPP1 blockade, detected by ELISA (*n* = 3; **** *p* < 0.0001). (**C**) Relative mRNA expression of S100A6 in ECs isolated from the co-culture system, determined by qRT-PCR (ns, no significant difference). (**D**) Relative mRNA expression of S100A6 in MAC cells isolated from the co-culture system, determined by qRT-PCR (*n* = 3; ** *p* < 0.01). (**E**) Western blot analysis and quantitative analysis of S100A6 protein levels in PANC-1 cells with or without recombinant S100A6 treatment. GAPDH was used as the internal loading control (*n* = 3; ** *p* < 0.01). (**F**) Schematic of the SPP1 loss- and gain-of-function co-culture experiment: macrophages transfected with control or SPP1-targeting siRNA were co-cultured with control endothelial cells, and S100A6 induction was assessed with or without anti-CD44 blockade or recombinant SPP1 (rSPP1) rescue. (**G**) Relative mRNA expression of S100A6 in the co-culture system across the indicated groups (Ctrl, si-SPP1, Ctrl + anti-CD44, Ctrl + IgG, si-SPP1 + rSPP1, si-SPP1 + rSPP1 + anti-CD44), determined by qRT-PCR (*n* = 3; **** *p* < 0.0001). (**H**) Cell viability of PANC-1 cells treated with S100A6 or control, measured by CCK-8 assay at the indicated time points (*** *p* < 0.001 at 96 h). (**I**) Representative immunofluorescence images of EdU staining (red) showing proliferating PANC-1 cells. Cell nuclei were counterstained with Hoechst 33342 (blue). Scale bar = 100 μm. (**J**) Quantification of the percentage of EdU-positive cells (**** *p* < 0.0001). (**K**) Representative images (**left**) and quantitative analysis (**right**) of colony formation assay for PANC-1 cells (*n* = 3; ** *p* < 0.01). (**L**) Cell viability of BxPC-3 cells treated with S100A6 or control, measured by CCK-8 assay (* *p* < 0.05 at 96 h). (**M**) Cell viability of MIA PaCa-2 cells treated with S100A6 or control, measured by CCK-8 assay (** *p* < 0.01 at 96 h). (**N**) Representative images of migrated MIA PaCa-2 cells in a Transwell migration assay stained with crystal violet. Scale bar = 100 μm.

We further verified the regulatory effect of the SPP1-CD44 axis on S100A6 expression using an EC-MAC co-culture system. ELISA results of the co-culture supernatant demonstrated that CD44 blockade exerted no significant effect on S100A6 protein levels under exogenous recombinant SPP1 stimulation, whereas SPP1 neutralizing antibody blockade significantly reduced S100A6 protein concentration under exogenous recombinant CD44 stimulation (Figure 6A,B). qRT-PCR analysis of sorted cell populations demonstrated that SPP1 blockade did not cause significant changes in S100A6 mRNA levels in ECs (Figure 6C; ns, no significant difference), but markedly downregulated S100A6 mRNA expression in MAC cells (Figure 6D; *p* < 0.01), indicating that SPP1-CD44 signaling mainly modulates S100A6 production in macrophages. To provide more direct functional evidence that activation of the SPP1-CD44 axis is responsible for S100A6 induction, we performed loss- and gain-of-function experiments in the co-culture system (Figure 6F). siRNA-mediated knockdown of SPP1 in macrophages markedly reduced S100A6 mRNA expression, and CD44 blockade with a neutralizing antibody produced a comparable reduction, whereas an IgG isotype control had no significant effect (Figure 6G; *p* < 0.0001). Importantly, supplementation with recombinant SPP1 restored S100A6 expression in SPP1-knockdown co-cultures, and this rescue was abolished by anti-CD44 blockade (Figure 6G), demonstrating that macrophage-derived SPP1 acts through endothelial CD44 to induce S100A6 expression.

To investigate the functional role of S100A6 in PDAC, we treated PANC-1 cells with recombinant S100A6 protein. Western blot confirmed successful upregulation of S100A6 protein in treated cells (Figure 6E; *p* < 0.01). Functional assays demonstrated that S100A6 treatment significantly enhanced cell viability of PANC-1 cells (*p* < 0.001 at 96h; Figure 6H), increased the proportion of EdU-positive proliferating cells (*p* < 0.0001; Figure 6I,J), and enhanced colony formation (*p* < 0.01; Figure 6K). The pro-proliferative effect of S100A6 was validated in additional PDAC cell lines: S100A6 treatment significantly increased cell viability of BxPC-3 cells (*p* < 0.05 at 96 h; Figure 6L) and MIA PaCa-2 cells (*p* < 0.01 at 96 h; Figure 6M). Transwell migration assay further confirmed the promotive effect of S100A6 on MIA PaCa-2 cell migration (Figure 6N). These findings indicate that S100A6 directly stimulates PDAC cell proliferation and migration.

**Figure 7:**
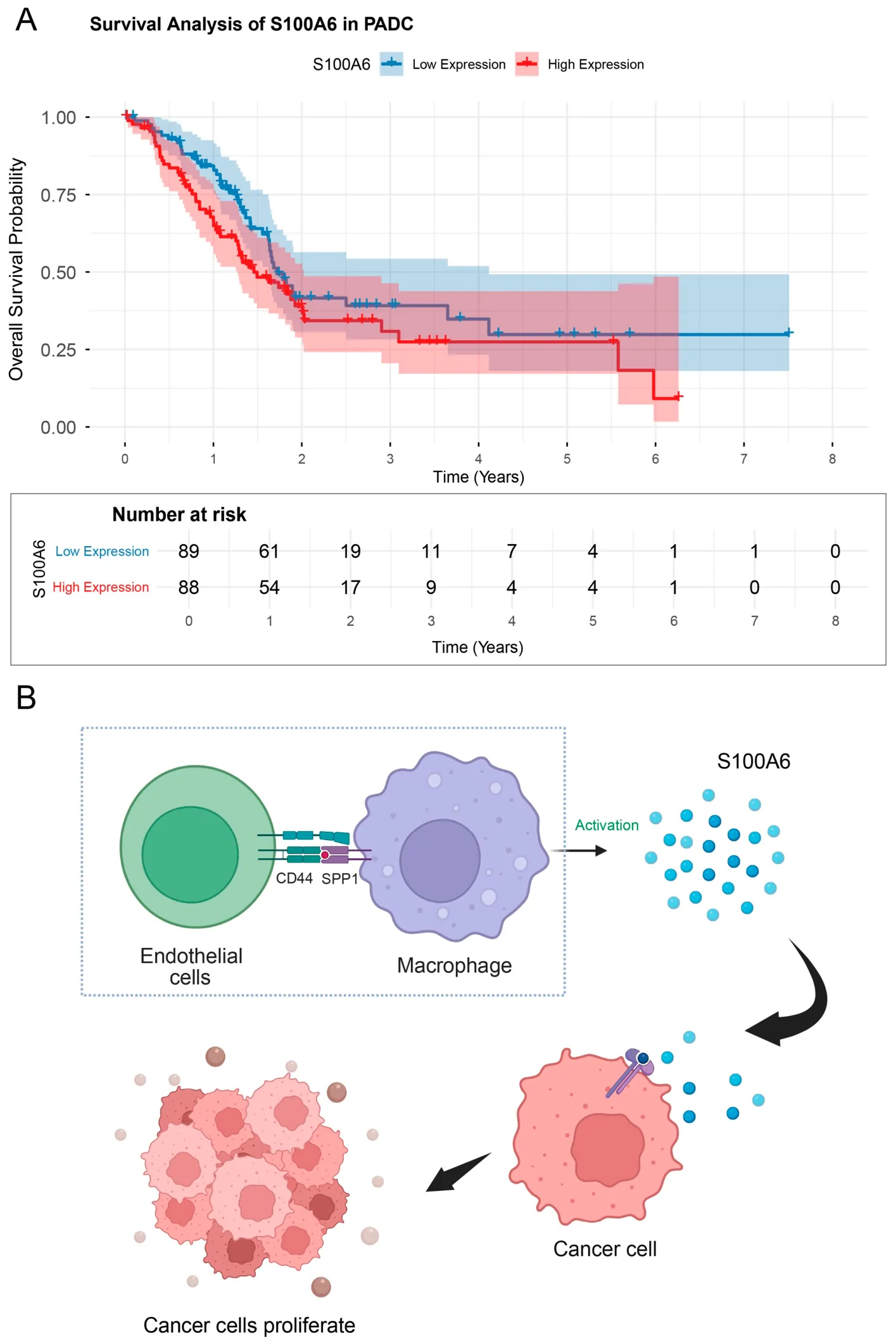
Prognostic value of S100A6 and proposed regulatory model. (**A**) Kaplan–Meier survival analysis of PDAC patients stratified by S100A6 expression (TCGA-PAAD cohort, *n* = 177). Patients with high S100A6 expression (red) exhibit significantly shorter overall survival compared to those with low expression (blue) (log-rank test, *p* < 0.05). Number at risk is shown below the survival curve. (**B**) Proposed model of S100A6-mediated regulation in PDAC. Our in vitro co-culture experiments demonstrate that S100A6 induction is functionally dependent on macrophage-derived SPP1 and endothelial CD44. In this model, Mac2-derived SPP1 binds to CD44 on EC0 and is proposed to trigger an as-yet-unidentified return signal that promotes S100A6 upregulation in Mac2; S100A6 may then act on PDAC cells to enhance proliferation and migration and thereby contribute to tumor progression.

To evaluate the clinical significance of S100A6, we performed survival analysis using the TCGA-PAAD cohort (*n* = 177). Patients with high S100A6 expression had significantly shorter overall survival compared to those with low expression (median survival: 14.2 vs. 25.6 months; log-rank *p* < 0.05; Figure 7A). Multivariate Cox regression further confirmed S100A6 as an independent prognostic factor (HR = 1.64, 95% CI = 1.03–2.61, *p* = 0.037). Cross-cancer analysis in the Human Protein Atlas revealed that S100A6 is most highly expressed in monocytes among immune cells (Figure S4A) and exhibits cancer-specific prognostic value, with significant association with poor survival only in PDAC and glioblastoma (Figure S4B–C).

Based on these findings, we propose a model wherein Mac2-derived SPP1 binds to CD44 expressed on EC0 endothelial cells, promoting S100A6 upregulation in the co-culture system, including in Mac2 macrophages. S100A6 then acts on PDAC cells to promote proliferation and migration, ultimately contributing to tumor progression (Figure 7B). Collectively, these data identify S100A6 as a key mediator of EC0–Mac2 crosstalk and a potential therapeutic target in PDAC.

## 4. Discussion

This study provides a comprehensive analysis of the heterogeneous landscape of endothelial and macrophage subpopulations in pancreatic ductal adenocarcinoma (PDAC), identifying an EC0–Mac2 interaction axis in which Mac2-derived SPP1 signals through CD44 expressed on EC0 and revealing S100A6 as a downstream effector of this axis that promotes PDAC progression. These findings demonstrate that crosstalk between stromal and immune cells in the PDAC microenvironment contributes to tumor progression, and they nominate several molecules for future functional investigation.

The identification of EC0 as a dominant endothelial subpopulation in PDAC (78.2% of total endothelial cells) marks a significant advance in our understanding of tumor vascular heterogeneity. EC0’s enrichment in PDAC, compared with 63.5% in adjacent normal tissues, aligns with its pro-angiogenic phenotype characterized by upregulation of VEGFA, ANGPT2, and metabolic regulators like MT-ATP6 and NDUFB8. This metabolic reprogramming—specifically the activation of “ATP synthesis coupled electron transport” and “mitochondrial respiratory chain complex assembly” pathways—supports the high energy demands of angiogenesis in the hypoxic tumor microenvironment. The observation that EC0 exhibits the highest “Hallmark_Angiogenesis” pathway scores further validates its central role in PDAC vascular remodeling, consistent with previous reports that tumor endothelial cells undergo dynamic phenotypic changes to facilitate neovascularization [29,30,31,32]. The expansion of EC0 in PDAC may therefore represent an adaptive response to the tumor’s need for increased nutrient and oxygen supply, making it a potential target for anti-angiogenic therapies.

Parallel analysis of macrophage subpopulations revealed Mac2 as a PDAC-enriched subset (32.1% vs. 0.1% in adjacent normal tissues) with a pro-tumorigenic transcriptional profile. Mac2’s upregulation of immunosuppressive markers, matrix remodeling enzymes, and anti-apoptotic regulators supports its role in creating an immune-privileged niche and promoting stromal desmoplasia, two hallmarks of PDAC. The finding that Mac2 is the primary source of SPP1 in PDAC is particularly noteworthy, as SPP1 has been implicated in multiple aspects of tumor progression, including macrophage recruitment, angiogenesis, and epithelial–mesenchymal transition [33,34,35,36]. The significant correlation between Mac2 expansion and EC0 enrichment suggests a potential coordinated remodeling of the endothelial and macrophage compartments in PDAC, potentially driven by reciprocal signaling loops that amplify pro-tumor phenotypes.

Cell–cell interaction analysis uncovered the SPP1-CD44 axis as a key mediator of EC0–Mac2 communication in PDAC. The observation that PDAC tissues exhibit an increase in total cell–cell interactions and a higher average interaction strength compared to adjacent normal tissues underscores the enhanced intercellular crosstalk in the tumor microenvironment. Network visualization confirmed that EC0 and Mac2 form a central communication hub, with SPP1-CD44 signaling accounting for the majority of their interactions. Our integrated analysis establishes the directionality of this interaction unambiguously: Mac2 macrophages, in which SPP1 is specifically upregulated (85% of Mac2 cells), are the source of the SPP1 ligand, whereas EC0 endothelial cells, which express the highest level of the principal SPP1 receptor CD44, are the recipient population (Figure 4A,C,D). This paracrine loop—where Mac2-derived SPP1 signals through CD44 on EC0—may contribute to the amplified angiogenic and immunosuppressive signals that promote tumor growth, although the return arm of the proposed cycle remains to be defined [37,38]. The strong positive correlations between SPP1 and CD44 expression further support the co-occurrence of this pathway in PDAC, and our functional perturbation and rescue experiments (Figure 6F,G) demonstrate that modulating SPP1-CD44 signaling directly affects S100A6 induction. Importantly, the in vitro co-culture experiments establish a functional dependency of S100A6 induction on the SPP1-CD44 interaction but do not identify the molecular step by which CD44 activation in EC0 signals back to Mac2 to upregulate S100A6; this return-signaling mechanism remains speculative and warrants further investigation.

The proposed role of S100A6 as a candidate downstream effector of the SPP1-CD44 axis adds another layer of complexity to PDAC progression. S100A6, a calcium-binding protein, was significantly upregulated in PDAC at both mRNA and protein levels, with high expression predicting poor patient survival [39,40,41]. Previous studies have reported S100A6 in PDAC ductal cells; our single-cell data suggest that, in addition, a macrophage subpopulation (Mac2) expresses S100A6 at the transcript level. The relative functional contribution of tumor-derived vs. macrophage-derived S100A6 remains to be determined. Functional assays demonstrated that S100A6 promotes PANC-1 cell proliferation and migration, consistent with its reported roles in cancer cell cycle regulation and cytoskeletal remodeling. The positive correlations between S100A6 and both SPP1 and CD44 expression, together with our new loss- and gain-of-function experiments showing that SPP1 knockdown reduces S100A6 expression and that recombinant SPP1 rescues it in a CD44-dependent manner (Figure 6F,G), demonstrate that SPP1-CD44 signaling induces S100A6 expression in the co-culture system. Whether S100A6 is the primary mediator of cancer cell effects in vivo remains to be determined. The observation that monocytes express S100A6 in the Human Protein Atlas is consistent with the possibility that their differentiated progeny, including Mac2 macrophages, may also express this gene. This raises the possibility that S100A6 could serve as a biomarker for identifying patients with active SPP1-CD44 signaling, as well as a therapeutic target for blocking the downstream effects of this pathway, but its therapeutic potential requires extensive further validation.

The clinical relevance of these findings is further supported by the observation that S100A6 expression is specific to PDAC and glioblastoma in terms of prognostic value, as shown in survival analysis across multiple cancer types [41,42,43,44]. This cancer-specific association suggests that S100A6 may play a unique role in tumors with dense stromal infiltration and high angiogenic activity, such as PDAC. Additionally, the enrichment of S100A6 in monocytes and macrophages—immune cells abundant in the PDAC microenvironment—highlights the potential for cell type-specific investigations targeting strategies that spare normal tissues.

Collectively, these results paint a detailed picture of how EC0 and Mac2 subpopulations drive PDAC progression through a signaling network centered on the SPP1-CD44 axis and its downstream effector S100A6. By demonstrating that EC0–Mac2 crosstalk enhances angiogenesis, immune suppression, and cancer cell proliferation, this study identifies multiple potential points of intervention. The in vitro co-culture data demonstrate that S100A6 induction is functionally dependent on SPP1-CD44 signaling, whereas the proposed EC0-to-Mac2 return-signaling step within this network remains hypothetical; further mechanistic dissection and in vivo validation will therefore be valuable to fully establish causality. Targeting EC0’s angiogenic phenotype, Mac2’s pro-tumor polarization, the SPP1-CD44 signaling axis, or S100A6’s downstream effects could each disrupt critical pathways in PDAC progression. Moreover, the strong correlations between these molecules and patient survival suggest they warrant further evaluation as valuable prognostic biomarkers, enabling more personalized approaches to PDAC management pending independent validation.

In conclusion, this study identifies EC0 and Mac2 as cell subpopulations that are enriched in PDAC and correlated with each other. Integrated single-cell and bulk analysis reveals an SPP1-CD44 communication axis between these subsets, in which Mac2-derived SPP1 signals through CD44 expressed on EC0 and is required for S100A6 induction in the co-culture system. Our in vitro data show that exogenous S100A6 promotes PDAC cell proliferation and migration. These findings are consistent with the EC0–Mac2–S100A6 network contributing to PDAC progression: the perturbation and rescue experiments demonstrate a functional dependency within the in vitro co-culture system. Together, the data provide a framework for future mechanistic and therapeutic studies. These findings provide new insights into the dynamic interplay between stromal and immune cells in PDAC and offer promising targets for therapeutic intervention. By disrupting these critical interactions, we may be able to halt tumor growth, restore immune surveillance, and improve outcomes for patients with this devastating disease.

## 5. Conclusions

In conclusion, this study provides an integrated single-cell transcriptomic analysis of endothelial and macrophage subpopulations in pancreatic ductal adenocarcinoma (PDAC). We identified an EC0 endothelial subset and a Mac2 macrophage subset that are enriched in PDAC and exhibit enhanced communication via the SPP1-CD44 axis, in which Mac2-derived SPP1 signals through CD44 expressed on EC0. In the in vitro co-culture system, S100A6 induction was dependent on SPP1 and endothelial CD44, as supported by SPP1 knockdown, CD44 blockade, and recombinant SPP1 rescue experiments. Exogenous S100A6 was further shown to promote PDAC cell proliferation and migration in vitro. These findings identify the EC0–Mac2–S100A6 network as a contributor to PDAC progression. Our work provides a framework for future mechanistic studies and suggests that components of this interaction axis warrant further exploration as potential prognostic biomarkers or therapeutic targets, pending rigorous in vivo validation.

## Data Availability

The original data presented in this study are openly available in the NCBI Gene Expression Omnibus (GEO) and the CNCB database. Accession numbers: GSE155698, GSE197177, GSE212966, and CRA001160. The TCGA-PAAD bulk RNA-seq and clinical data are available from the TCGA Data Portal (https://portal.gdc.cancer.gov/, accessed on 8 October 2026).

## Supplementary Materials

The following supporting information can be downloaded at https://www.mdpi.com/article/10.3390/cells15201844/s1: Figure S1. Validation of endothelial and macrophage markers across datasets. Figure S2. Differential gene expression and angiogenic pathway activity in endothelial subpopulations. Figure S3. Characterization of signaling pathways and interaction strength between endothelial and macrophage subpopulations. Figure S4. S100A6 expression across immune cell populations and cancer types.

## Abbreviations

The following abbreviations are used in this manuscript:

PDAC	Pancreatic ductal adenocarcinoma
TME	Tumor microenvironment
EC	Endothelial cell
MAC	Macrophage
