# Supplementary material for: SPP1-CD44-Mediated Crosstalk Between Endothelial and Macrophage Subpopulations Promotes Pancreatic Ductal Adenocarcinoma Progression Through S100A6 Upregulation
Source: Cells. 2026 Oct 11;15(20):1844. doi: 10.3390/cells15201844 (PMC13703074; doi:10.3390/cells15201844)
Supplement: Supplementary file 1 [file cells-15-01844-s001.zip › cells-4547552-supplementary.pdf]

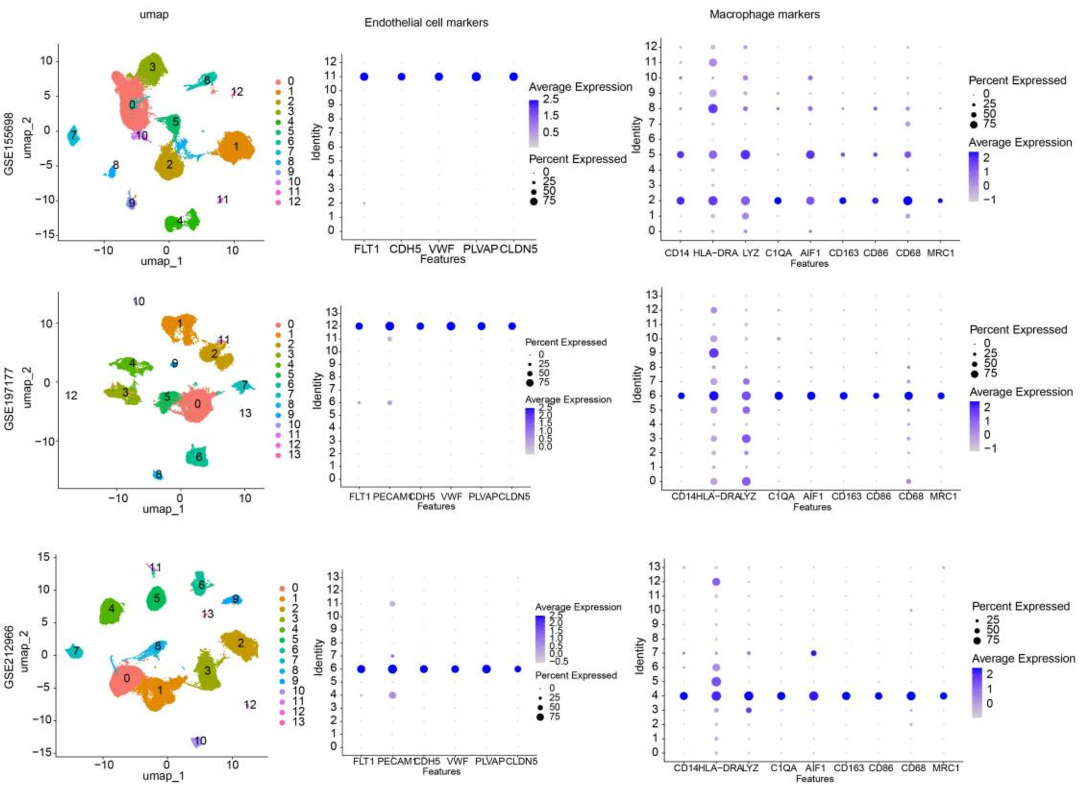

Figure S1. Validation of endothelial and macrophage markers across datasets. UMAP visualization of immune and stromal cell populations in three independent datasets (GSE155688, GSE191777, GSE212966) with color-coded clusters (left panels). Middle panels: Dot plots showing expression of canonical endothelial markers (FLT1, PECAM1/CD31, CDH5, VWF, PLVAP, CLDN5) across clusters, with dot size representing the percentage of cells expressing the marker and color indicating average expression level. Right panels: Dot plots showing expression of macrophage markers (CD14, HLA-DRA, LYZ, ITGAX/CD11c, AF1, CD163, CD68, CD86, MRC1/CD206) across clusters. Endothelial and macrophage populations exhibit consistent marker expression patterns across all datasets.

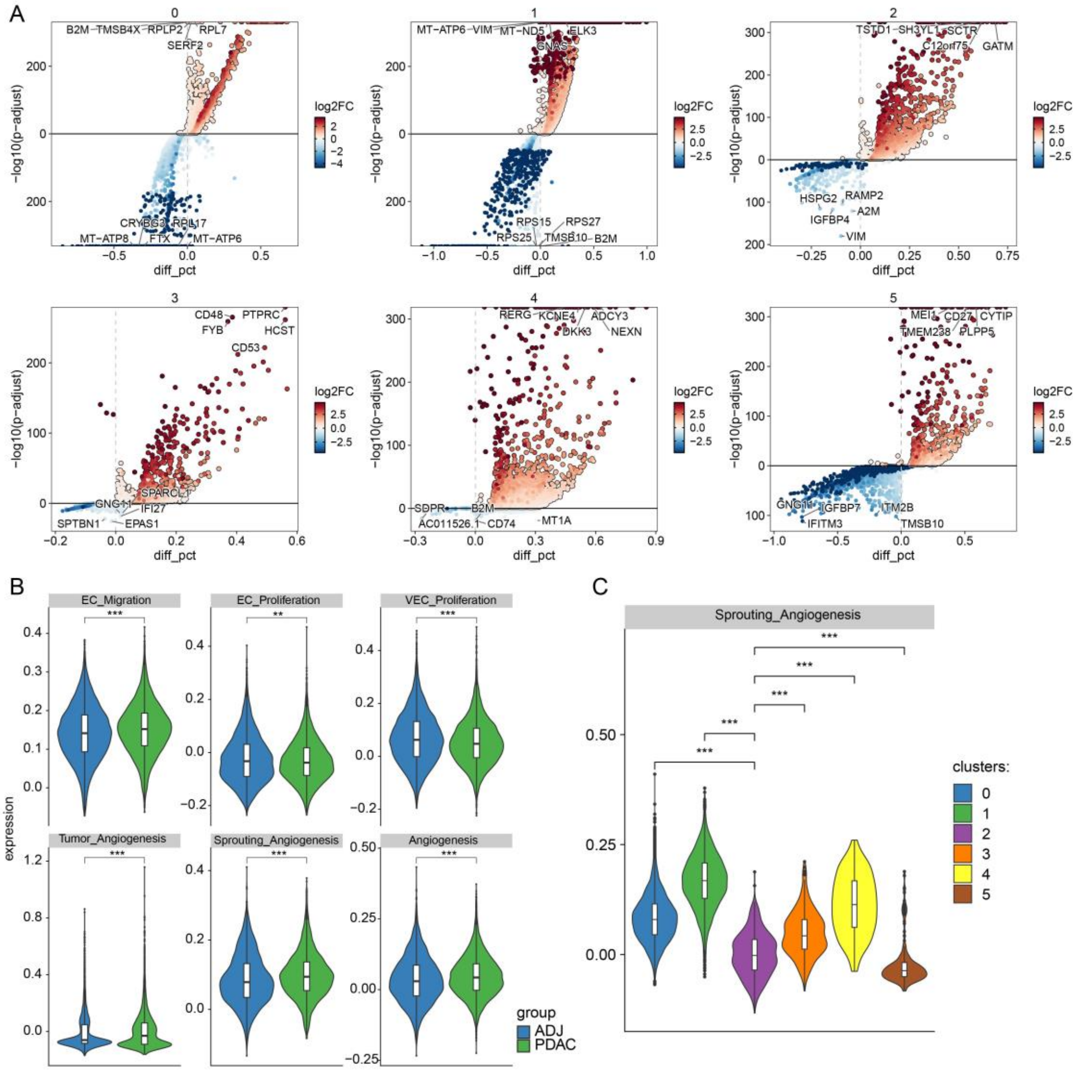

Figure S2. Differential gene expression and angiogenic pathway activity in endothelial subpopulations. (A) Volcano plots showing differentially expressed genes between EC0 and other endothelial subpopulations (clusters 1–5). Red dots indicate upregulated genes (log2FC > 0.25, adjusted $p < 0.05$), blue dots indicate downregulated genes (log2FC < -0.25, adjusted $p < 0.05$), and gray dots indicate non-significant genes. Selected angiogenesis-related genes are labeled. (B) Violin plots comparing pathway activity scores for endothelial function-related pathways ("EC Migration," "EC Proliferation," "Tumor_Angiogenesis," "Sprouting_Angiogenesis," "Angiogenesis") between adjacent normal (ADJ) and PDAC tissues. p<0.01,* **p<0.001,****p<0.0001 (two-tailed Student's t-test). (C) Violin plots showing "Sprouting_Angiogenesis" pathway activity scores across endothelial subpopulations (EC0–EC5) in PDAC. **p<0.001,****p<0.0001 (one-way ANOVA with Tukey's post-hoc test).

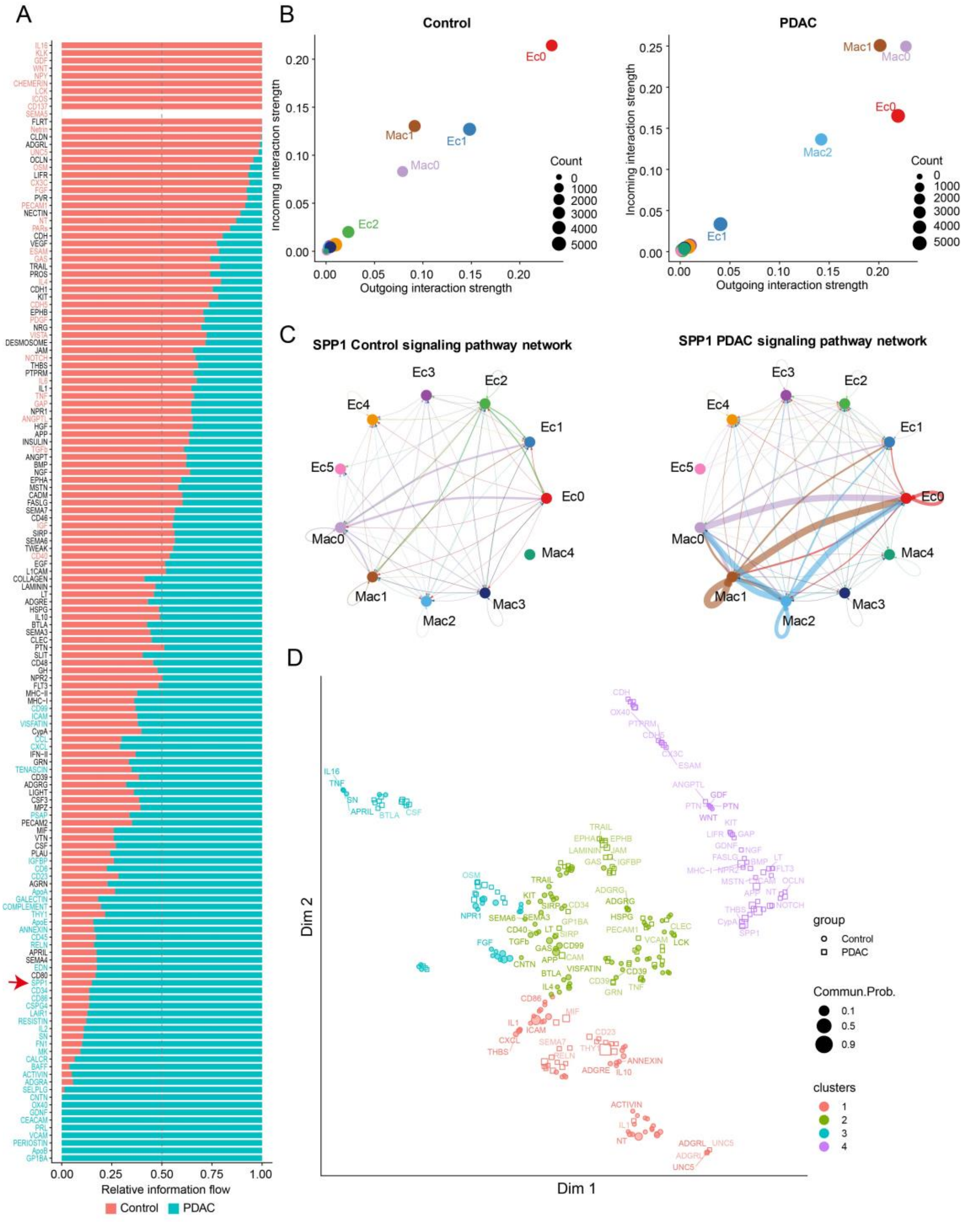

Figure S3. Characterization of signaling pathways and interaction strength between endothelial and macrophage subpopulations. (A) Bar plot showing relative information flow of signaling pathways in control (ADJ, red) and PDAC (blue) tissues. The SPP1 pathway exhibits the highest information flow in PDAC (arrow). (B) Scatter plots comparing outgoing vs. incoming interaction strength of endothelial and macrophage subpopulations in control (left) and PDAC (right) tissues. Dot size represents cell count. EC0 (red) shows significant interaction strength in PDAC. (C) Network diagrams visualizing SPP1 signaling pathways in control (left) and PDAC (right) tissues. EC0 (red node) and Mac2 (blue node) demonstrate enhanced SPP1-mediated communication in PDAC (thickened edges). (D) Dimensionality reduction plot (t-SNE) showing signaling pathway distribution in control (circle) and PDAC (square) tissues. Color indicates subpopulation; dot size represents communication probability.

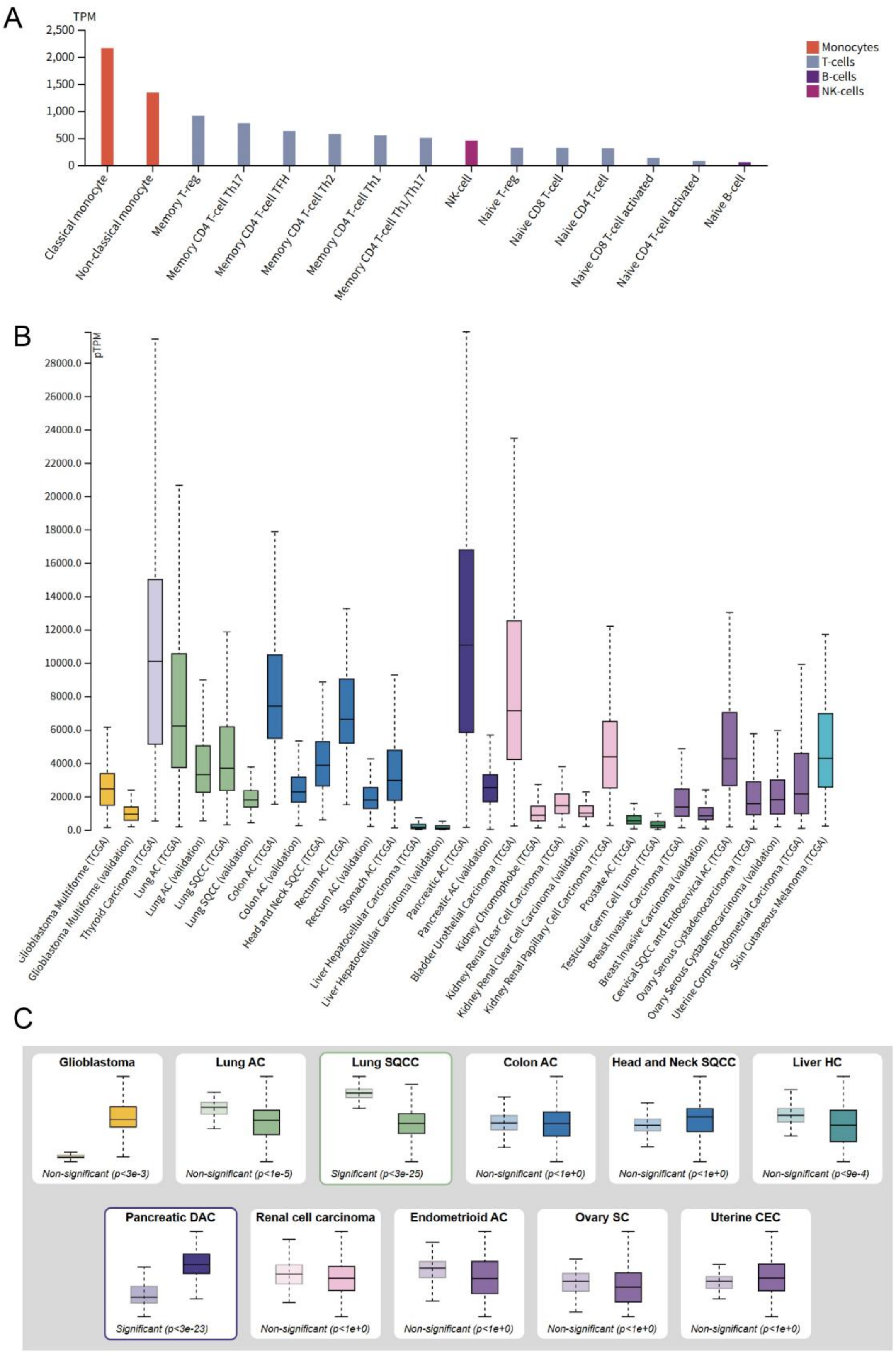

Figure S4. S100A6 expression across immune cell populations and cancer types. (A) Bar plot

showing S100A6 mRNA expression (TPM) in human immune cell populations from the Human Protein Atlas. Monocytes exhibit the highest S100A6 expression among immune cells. (B) Bar plot comparing S100A6 protein expression (normalized spectral counts) across different human cancer types. Pancreatic adenocarcinoma (PDAC) shows intermediate expression relative to other malignancies. (C) Survival analysis of S100A6 across cancer types from TCGA. High S100A6 expression correlates with poor prognosis in PDAC ($p<0.05$) and glioblastoma, but not in other cancer types analyzed.
